# Microablative Fractional Radiofrequency as a Therapeutical Option for Genitourinary Syndrome of Menopause: Perspectives

**DOI:** 10.3389/frph.2021.779421

**Published:** 2021-12-07

**Authors:** Márcia Farina Kamilos, Ana Paula Ferreira Costa, Ayane Cristine Alves Sarmento, José Eleutério, Ana Katherine Gonçalves

**Affiliations:** ^1^Department of Gynecology, Hospital Heliópolis, São Paulo, Brazil; ^2^Postgraduate Program in Health Sciences, Federal University of Rio Grande Do Norte (UFRN), Natal, Brazil; ^3^Department of Obstetrics and Gynecology, Federal University of Ceará, Fortaleza, Brazil; ^4^Department of Obstetrics and Gynecology, Federal University of Rio Grande Do Norte (UFRN), Natal, Brazil

**Keywords:** genitourinary syndrome of menopause, urinary incontinence, radiofrequency, dyspareunia, atrophy

## Abstract

The genitourinary syndrome in menopause can occur at different stages of life, with different causes or triggering factors, such as prolonged use of antiestrogens, chemotherapy, radiotherapy, and extensive vaginal surgeries, which can alter vascularization, hydration, collagen quality, and tissue elasticity. Despite hormonal therapy being considered the best evidenced treatment for genitourinary syndrome of menopause (GSM), there are limitations concerning the latter. Thus, alternative, complementary, or even substitutive treatments have emerged, such as energy use, promoting thermal tissue stimulation to improve tropism. Due to its practicality and feasibility, the micro ablative fractional radiofrequency (MAFRF) has gained space among these energies. It uses high-frequency electromagnetic waves and promotes thermal micro points in the superficial and deep dermis. The safety of these energies limits thermal action laterality and depth. Laterally, it is essential for an adequate regenerative effect without scarring marks or sequelae; the appropriate depth is important for stimulating the obligatory tissue repair response with the production and reorganization of collagen, elastic fibers, increased vascularization and hydration, and the consequent improvement in tropism. In gynecology, the MAFRF is used with therapeutic indication and functional improvement; it is applied to the entire length of the vaginal walls, the vulvar vestibule, urethral meatus, labia minora, clitoris prepuce, labia majora, perineum, and perianal region. The MAFRF has been proved to be an effective and safe treatment for GSM, with long-lasting effects, significantly reducing symptoms and improving vaginal tropism. This review aims to analyze the MAFRF as a non-hormonal therapeutic option for GSM.

## Introduction

The genitourinary syndrome in menopause encompasses symptoms such as dyspareunia, burning and vulvovaginal dryness, urinary urgency, and stress urinary incontinence (SUI), which can occur at different stages of life with different causes or triggering factors, the most frequent being the decrease in estrogen (1, 2). The prolonged use of antiestrogen drugs, chemotherapy, radiotherapy (RT), and extensive vaginal surgeries can alter the vascularization, hydration, collagen quality, and elasticity of the genital tissue (1–3).

Hormone therapy is considered extremely recommendable for genitourinary syndrome of menopause (GSM) with a high level of evidence behind it. However, complementary or even substitutive treatments have emerged, such as energy use, promoting thermal tissue stimulation to improve tropism. Among energies, the MAFRF has gained an exciting space due to its practicality and feasibility in all spheres of providing healthcare services, with safety and efficacy (4).

The MAFRF devices use thermal energy generated by an electrical current rather than laser light and are not subject to diffraction or absorption by epidermal chromophores, allowing them to be safely used on all skin types. Fractional technology has replaced ablative technology, with the advantages of reducing complications such as skin hyperpigmentation, a shorter recovery period, lasting effects, and the possibility of reapplication (5, 6).

In gynecology, MAFRF is used as a therapeutic indication and functional improvement. It is applied to the entire length of the vaginal walls, the vulvar vestibule, urethral meatus, labia minora, clitoris prepuce, interlabial creases, and the inner surface of the labia majora, perineum, and perianal region. The MAFRF has been proved to be an effective and safe treatment for GSM, with long-lasting effects, significantly reducing symptoms and improving vaginal tropism.

## Therapeutical Perspectives of Microablative Fractional Radiofrequency

**Figure 1:**
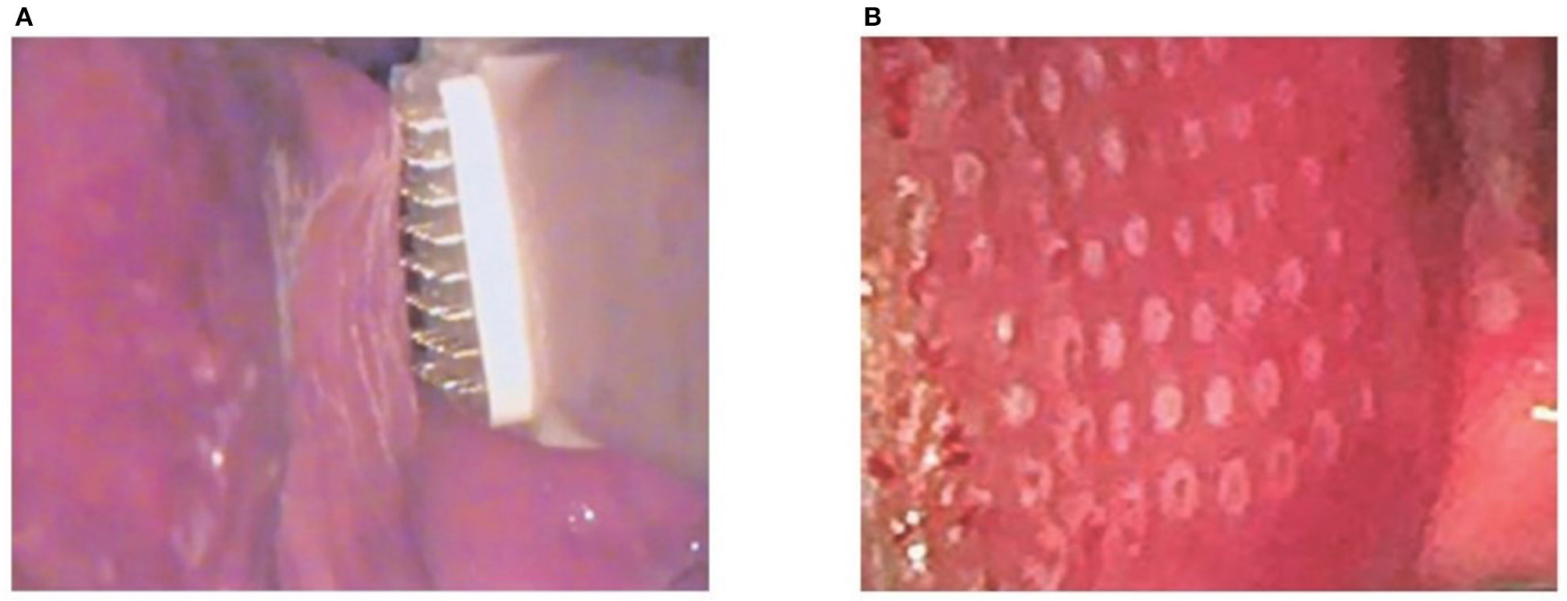
The electrode is parallel and lightly touching the mucosa and the subsequent microablations. Adapted from Sarmento et al. (7). **(A)** Radio frequency application. **(B)** Vaginal mucosa after radiofrequency application.

**Figure 2:**
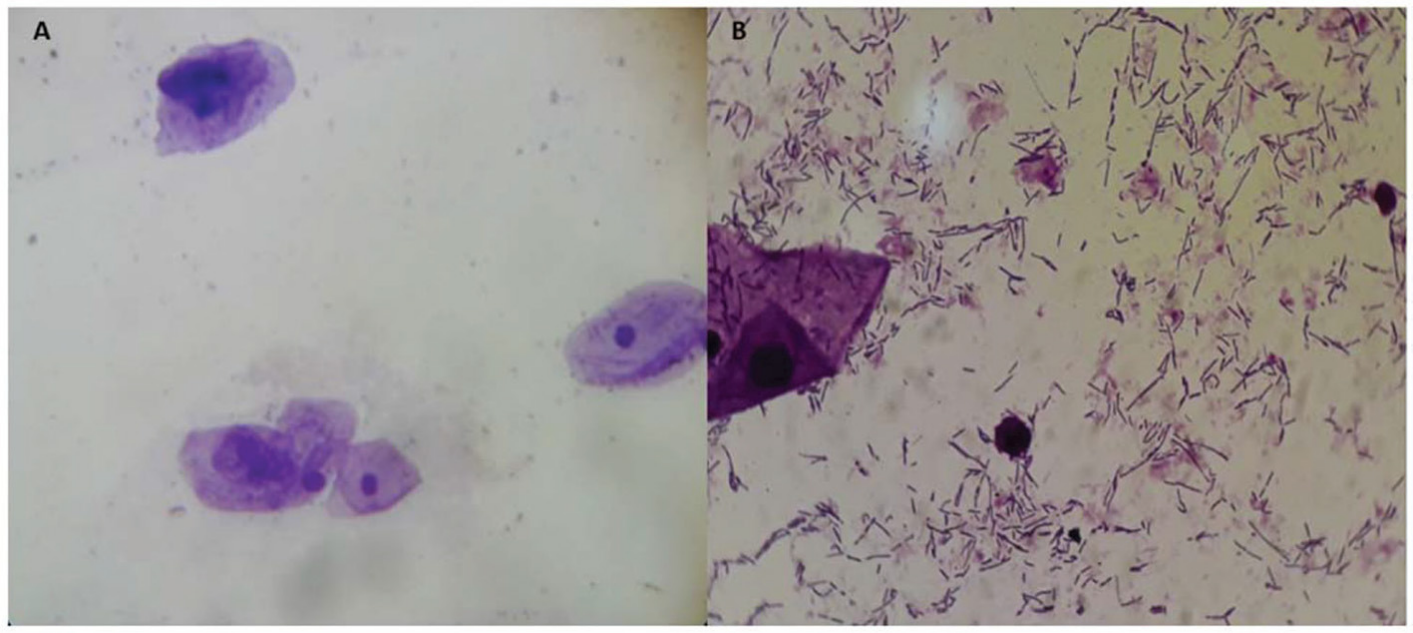
Before **(A)** and after treatment **(B)**. Adapted from Sarmento et al. (7).

The first pilot study on vaginal MAFRF (Linly™ Loktal Medical Electronics) evaluates the clinical response of 14 patients with symptoms of GSM after application of MAFRF in the vagina and vaginal introitus. Since the application is carried out under direct vision and using a vaginal speculum, treatment along the vaginal walls is facilitated, preventing shots from overlapping. For the procedure, the Wavetronic 6000 Touch device was used with the Megapulse HF FRAXX system (Loktal Medical Electronics, São Paulo, Brazil), equipped with an electronic circuit of energy fractionation, connected to a vaginal pen with 64 microneedles, 200 μ in diameter and 1 mm in length, mounted on a Teflon body and divided into an eight-column matrix with eight needles each (Figures 1A,B). The MAFRF effectively treated vaginal dryness and dyspareunia symptoms, eliminating the use of lubricants during the observed period [(6) Figure 2].

Another more robust study analyzed the impact of MAFRF on the vaginal health, microbiota, and cellularity of postmenopausal women, suggesting that MAFRF treatment is well-tolerated by women and leads to significant improvement in the vaginal microenvironment. Therefore, radiofrequency (RF) can be used to treat the vaginal symptoms of GSM. The therapy restored the vaginal balance, as would usually be expected with sufficient estrogen levels (Figures 2A,B). The predominance of *Lactobacillus* species and acidic pH of the vaginal fluid achieved after RF therapy could protect postmenopausal women from vaginal infections, inflammation, and infections of the urogenital tract (7).

A randomized clinical trial performed with MAFRF for stress urinary incontinence (SUI) aimed to compare the effect of MAFRF and pelvic floor muscle training (PFMT) against the combination of both therapies (MAFRF + PFMT) in the SUI and on genitourinary syndrome (GSM), including 117 climacteric women with SUI. Urinary scores improved significantly in all three groups posttreatment (*p* <0.001) with a higher improvement in the RF + PFMT group (*p* = 0.002). Vaginal symptoms showed an incremental improvement in MAFRF (*p* <0.007), and vaginal laxity showed a similar improvement (*p* = 0.323). The Vaginal Health Index score was higher in RF and RF + PFMT groups. Sexual function improved in RF and PFMT. The association between RF and PFMT showed significant improvement in the SUI symptoms assessed by the questionnaire, the vaginal symptoms and dryness showed improvement in the RF treatment, and the vaginal laxity showed similar improvement. The combination of RF and PFMT in sexual function did not show benefits superior to those achieved by the therapies alone (8).

Another study, a review of light and energy-based therapeutics for GSM, used by gynecologists, urogynecologists, dermatologists, and plastic surgeons, observed that the use of a CO^2^ laser, Erbium laser, and RF promoted improvement of SUI symptoms through induction of 30% constriction in the suburethral area of the vagina, associated with neocollagenesis, elastogenesis, and angiogenesis (9, 10).

Another application of MAFRF studied was in vulvar sclerosus lichen to assess the clinical response to and the histomorphometric effects of MAFRF in women with symptomatic vulvar lichen sclerosus (VLS). After two to three sessions, most of the participants became asymptomatic, with nearly 40% of the participants reporting complete remission of symptoms. The improvement was rated as moderate or higher and persisted for 11 months (range, 7–16 months), on average after the treatment. The study showed that it is an effective and safe treatment for symptomatic vulvar lichen sclerosus, with long lasting effects, particularly to reduce pruritus and burning sensation,; type III collagen concentration significantly increased in the proportion of type III to type I collagen after treatment and was associated with important symptom improvement. This suggests that MAFRF may improve “elasticity” or “plasticity” by increasing the number of thinner type III collagen fibers (11).

## Discussion

High-frequency RF devices (4 MHz) associated with different types of waves in the same device, such as continuous linear, pulsed, and fractional microablative for skin and vaginal mucosa, provided multi-application in gynecology (6). Continuous linear RF is used in excisional and ablative procedures in the cervix, vagina, and vulva. Pulsed RF allows control of the depth of the thermal effect, being used to destroy vaginal and vulvar diseases, such as intraepithelial lesions, condylomas, among others. MAFRF is the most recent modality introduced in some devices and used in several medical specialties, such as dermatology, urogynecology, gynecology, plastic surgery, and even proctology (6, 11, 12).

The MAFR is an effective and safe treatment for GSM, significantly reducing symptoms and improving vaginal trophism, with the advantage of lasting effects, long-term maintenance. It can be used in association with hormone therapy or as a substitute, especially in cases of contraindication to the use of hormone therapy or the desire of the patient, as a non-hormonal therapeutic option for GSM and other situations that cause vulvovaginal atrophy (12, 13).

## Conclusion

More comparative studies between different radiofrequency devices and other energy devices such as laser and micro-focused ultrasound are needed to establish more specific protocols and assess the long-term safety of tissue effects. The perspective of MAFRF is an effective and safe treatment to GSM due to showing reduction of the symptoms and improvement of tropism, with long-lasting results.

## Author Contributions

MK and AG conceived and designed the study and prepared the figures. MK, AG, and AC drafted and revised the article where appropriate. AG, JE, and AS carried out the final revision of the manuscript. All authors contributed to the article and approved the submitted version.

## Conflict of Interest

The authors declare that the research was conducted in the absence of any commercial or financial relationships that could be construed as a potential conflict of interest.

## Publisher's Note

All claims expressed in this article are solely those of the authors and do not necessarily represent those of their affiliated organizations, or those of the publisher, the editors and the reviewers. Any product that may be evaluated in this article, or claim that may be made by its manufacturer, is not guaranteed or endorsed by the publisher.
